# Effectiveness of Different Etching Agents on Enamel Surface and Shear Bond Strength: An In Vitro Evaluation

**DOI:** 10.7759/cureus.54008

**Published:** 2024-02-11

**Authors:** Bharvi Jani, Alap Shah, Chetan Shankar, Siddharth Revankar, Hetanshi S Shukla, Karthikeyan Ramalingam

**Affiliations:** 1 Orthodontics and Dentofacial Orthopedics, Karnavati School of Dentistry, Karnavati University, Gandhinagar, IND; 2 Orthodontics and Dentofacial Orthopedics, Vydehi Institute of Dental Sciences and Research Centre, Bengaluru, IND; 3 Orthodontics and Dentofacial Orthopedics, Maratha Mandal's Nathajirao G. Halgekar Institute of Dental Sciences and Research Centre, Belagavi, IND; 4 Oral Pathology and Microbiology, Saveetha Dental College and Hospitals, Saveetha Institute of Medical and Technical Sciences, Saveetha University, Chennai, IND

**Keywords:** etching depth, etching pattern, universal testing machine, citric acid, phosphoric acid, hydrofluoric acid, acid etching, orthodontics, scanning electron microscopy, shear bond strength

## Abstract

Background

Enamel etching is of utmost importance during the orthodontic bonding procedure. Phosphoric acid, hydrofluoric acid, and citric acid are used in specific concentrations to create surface irregularities on enamel surfaces, enhancing the bond strength of the orthodontic attachment. Therefore, it is essential to evaluate the type of etchant for reliable orthodontic bracket bonding with minimal damage to the enamel surface.

Aims and objectives

This study aimed to investigate the morphological changes on the enamel surface after treatment with different surface etchants, assess the depth of penetration, and evaluate the shear bond strength (SBS) of orthodontic brackets.

Materials and methods

One hundred and one extracted premolar teeth were used to investigate morphological changes on the enamel surface treated with 37% phosphoric acid, 11% hydrofluoric acid, and 20% citric acid. It was evaluated on a scanning electron microscope (Jeol Scientific Equipment, Jeol Limited, Akishima, Japan), and the SBS of brackets on enamel treated with different etching agents was evaluated using an Instron Universal Testing Machine (UTM; Instron Model: 5982, Universal Testing Systems, Norwood, MA). Group A had 60 test samples. Group B had 40 test samples. One control without any acid etching was used in both groups. Subgroup A1 (n = 30) was evaluated for surface characteristics of acid-etched enamel. Subgroup A2 was assessed for the penetration depth of various etchants. Group B (n = 40) was tested for SBS. The results were tabulated and analyzed using IBM SPSS Statistics, version 20.0 (IBM Corp., Armonk, NY). Post hoc Tukey HSD test and one-way analysis of variance were used to assess SBS and penetration depth of etchants (P ≤ 0.05). Pearson’s correlation test was used to correlate SBS, etching pattern, and penetration depth. The chi-square test was used to test the frequency of types of etching patterns.

Results

Intergroup correlations between etching depth, etching pattern evaluated on SEM, and SBS evaluated on the UTM showed a high statistical correlation between etching depth & SBS, etching depth & etching pattern, and SBS & etching pattern between A1, A2, and group B (P ≤ 0.001). A highly significant negative correlation between SBS & etching pattern (P = 0.42) was observed among intra-group correlation. Non-significant correlations were found between etching depth & SBS and etching depth & etching pattern within the 20% citric acid etch group (P = 0.370 and 0.141, respectively).

Conclusion

Penetration depth obtained was highest with 11% hydrofluoric acid, followed by 37% phosphoric acid and 20% citric acid. In addition, 11% hydrofluoric acid showed the highest bond strength. Acid etching showed better penetration depth and bond strength than control.

## Introduction

The micro-mechanical interlocking of the adhesive to the etched enamel surface and the bracket base determines the strength of orthodontic attachments. Bond failure during orthodontic treatment leads to the detachment of a bracket or any other bonded attachments. Bond failure is a common problem during clinical practice in orthodontics. To minimize bond failure rate, accurate bracket positioning is of utmost importance, as frequent rebonding increases treatment time [1].

The acid etching procedure produces micro-porosities by partial decalcification of the dental enamel surface. Among all the steps of orthodontic bracket bonding, the type of acid etchant and its concentration are important factors determining the retention of bonded attachments. Thus, acid etching has drawn a lot of attention from researchers [1,2]. Prolonged retention of the adhesive onto the enamel surface was observed when the adhesive was coated immediately after etching [2-4]. Bond failure can be prevented by the consistent prism-end structure on etched enamel. The prism formation and etching patterns can be visualized by scanning electron microscopy (SEM). The depth of the enamel etching can also be determined by SEM [5]. 

Every step in bonding brackets must be carried out with caution, as bond failures can be encountered routinely in clinical practice. The acids most commonly used for achieving micro-irregularities in the tooth structure are phosphoric acid, hydrofluoric acid, citric acid, polyacrylic acid, nitric acid, EDTA, maleic acid, etc. However, 37% phosphoric acid is the gold standard, and variable concentrations of other etchants have been tested in the literature. Disadvantages of acid etching include localized decalcification of enamel and risk of enamel fracture. However, controversy remains on the effectiveness of different dental etchants, the optimal etching duration, and the concentration of specific etchants [6,7].

In vitro shear bond strength (SBS) testing is a widely used method to assess the bond strength between orthodontic brackets and tooth enamel. Thus, research should be conducted to evaluate the difference in surface texture, depth of enamel tags, and SBS of bonded brackets when the dental enamel is treated with different surface etching agents. In this study, we have evaluated the etching pattern, penetration depth, and SBS of different etching agents utilizing SEM and a universal testing machine (UTM).

## Materials and methods

Our study was approved by the Institutional Ethical Committee of Karnavati School of Dentistry, vide letter number: KSDEC/21-22/Apr/017. Premolars are the most commonly extracted teeth for orthodontic purposes, and thus, this study was performed with extracted premolar teeth. Maxillary and mandibular premolars extracted for orthodontic treatment were used for investigation. The study included intact and fully erupted teeth, whereas the teeth with fluorosis, carious lesions, restorations, and enamel defects were excluded.

As two different parameters were assessed, based on power analysis, the sample size calculation was done. GPower 3.1 software was utilized (effect size = 0.282; α = 0.05; 1 − β = 0.80; N2/N1 = 1) for sample size estimation. One hundred samples were determined as the required quantity. One hundred and one extracted premolar teeth were divided into two groups, group A (n = 60) and group B (n = 40), to assess morphological characteristics of the enamel surface and determine SBS, respectively. One sample was used as a control in which no acid etching was performed. The control was included to evaluate and compare the surface characteristics of the untreated enamel surface with the etched enamel surface under SEM. The SEM study was done at Gujarat Industrial Development Corporation (GIDC), Gandhinagar, India, utilizing a scanning electron microscope (Jeol Scientific Equipment, Jeol Limited, Akishima, Japan).

The test for measurement of SBS was carried out at Ahmedabad Textile Industry’s Research Association (ATIRA), Ahmedabad, India. SBS evaluation was carried out on the Instron Universal Testing Machine (Instron Model: 5982, Universal Testing Systems, Norwood, MA).

Scanning electron microscopy (SEM)

The effects of etching agents on enamel tooth surfaces were evaluated in the laboratory through SEM. Prophylaxis of the enamel surface was performed using a rubber cup mounted to a handpiece at a slow speed for 10 seconds. It was rinsed with a water spray for 15 seconds and dried using an air spray for 10 seconds. The specimens were stored in distilled water at room temperature (37°C) to prevent dehydration [8]. Group A (n = 60) samples were further divided into two groups: A1 (n = 30) and A2 (n = 30). Group A1 was used for the evaluation of surface characteristics of the enamel tooth surface after the application of three different etching agents (citric acid, phosphoric acid, and hydrofluoric acid). The depth of penetration of three different etching agents into the enamel of the cross-sectioned teeth samples was seen in group A2. The penetration depth was measured in microns from a point on the outer enamel surface to the deepest etched point. To receive the three different etchings, these were again subdivided into three groups: x, y, and z, each containing 10 teeth.

**Figure 1:**
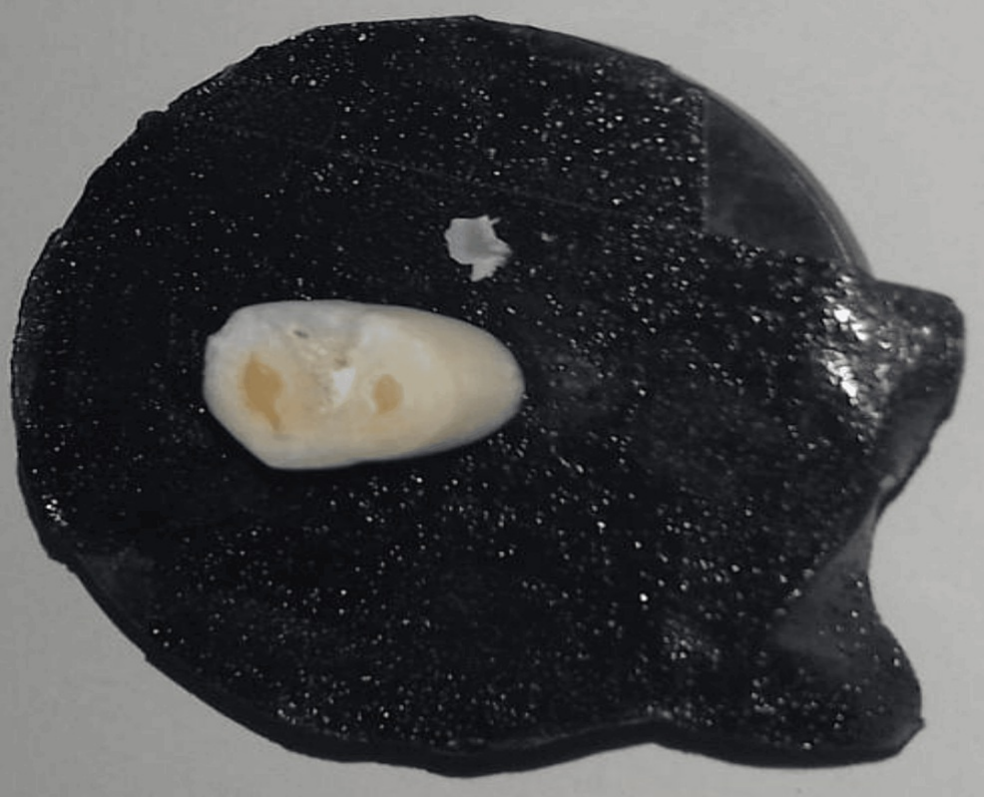
Cross-sectioned tooth sample mounted on an aluminum stub

A sputter coater (Polaron SC7610, Fisons Instruments, Sussex, UK) was utilized to apply the gold coating of test samples before SEM evaluation. Sample preparation for the SEM study was done to observe the morphological characteristics of the enamel tooth surface using three different etching agents (group A1). The teeth samples (N = 30) were prepared by sectioning the root of each tooth 2-3 mm below the cementoenamel junction (Figure 1).

Sample preparation was done to determine the penetration depth of three different etching agents into the enamel surface (n = 30). Cusp tips of the teeth were cross-sectioned with motorized diamond abrasives-coated disc burs along with water coolant spray to avoid damage to the enamel surface by heat generated (group A2). For improved visibility, samples were further polished with pumice slurry & rubber cup for 10 seconds before etching.

**Figure 2:**
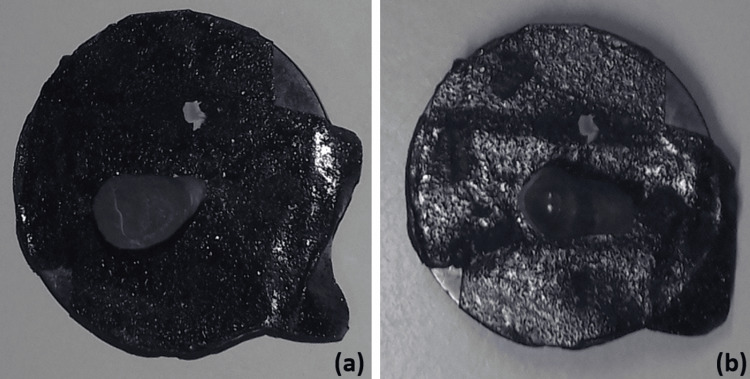
(a) The cross-sectioned tooth and (b) the buccal tooth surface with gold sputter coating

The buccal surface of each tooth was treated sequentially with the application of three different types of etchants. The etching was done at room temperature (37°C), and three different etching agents were applied sequentially on the buccal surface of teeth for 30 seconds, followed by rinsing and drying each sample for 15 seconds. The appearance of chalky white enamel was noted. The sectioned specimens were mounted, and the gold sputter coating was applied (Figure 2).

Following preparation, specimens were viewed at 10 kV of accelerating voltage, and images were taken under 100×, 1000×, and 5000× magnification for etching patterns and penetration depth. The etching patterns on the enamel surface observed were classified into five types, according to Silverstone et al. [5]. Type 1 has a honeycomb pattern with preferential dissolution of prism cores. Type 2 has a cobblestone pattern with preferential dissolution of prism peripheries. Type 3 has a random etch pattern that could not be connected to prism morphology. Type 4 shows enamel surface pitting. Type 5 shows a smooth enamel surface without any micro-irregularities. For evaluation of surface roughness, cross-sectional views of the specimens were examined at 100× magnification. A standard linear length of 100 μm was used to calibrate the dimensions within the 100× SEM. The measurement of the surface roughness of the etched enamel was performed using a tracing tool within the set length of 100 μm. 

Shear bond strength (SBS)

Group B was subdivided into I, II, III, and IV, with 10 teeth in each subdivision to evaluate SBS. After etching the enamel surface, a primer (Transbond XT, 3M Unitek, Monrovia, CA) was applied with the help of an applicator tip, and the light was cured. The adhesive (Transbond XT, 3M Unitek) resin composite was then applied to the bracket base, and the bracket placement was performed [9]. Using a Supra Blue LED curing light (Woodpecker i-LED, Guilin Woodpecker Medical Instruments, Guilin, China), specimens were light-cured considering the manufacturer’s instructions. A curing tip of 8 mm diameter at a light intensity of 1000 mW/cm^2^ and a wavelength of 420-480 nm was used with a standard 40-second cure time, 10 seconds proximally, 10 seconds occlusal, and gingivally [6]. The frequency of the light emitted was calibrated using an in-built light intensity meter before use. The indicator light indicates whether the output is sufficient, and the sensor receives the light tip. At 37 ± 1°C for 24 hours, all specimens were maintained in darkness until the adhesive gained optimal bond strength [10]. 

**Figure 3:**
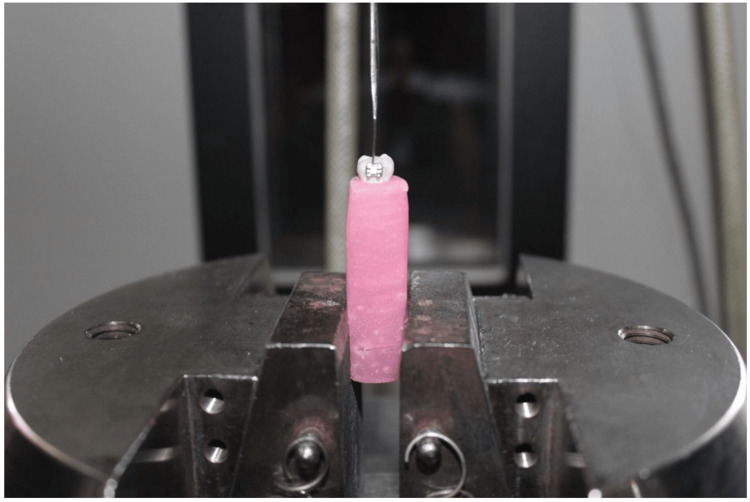
Picture showing the mounted sample on the Instron Universal Testing Machine

A digital vernier caliper (Baker Gauges, Pune, India) was used to determine the bracket base dimension (average sum of the widths and lengths of 10 brackets) with an accuracy of 0.01 mm. The load at which metal brackets were debonded was recorded in Newton. SBS was calculated using the following formula: SBS = load (N)/surface area (mm^2^) and was recorded in megapascals (MPa). The specimen was placed in the Instron Universal Testing Machine such that the base of the bracket was parallel to the applied debonding force (Figure 3).

At the interface of the composite and bracket base, a stainless-steel blade 1 mm thick with a 45° inclination at the tip was adapted. A shear debonding force was applied, and the direction of the force was perpendicular to the long axis of the specimens. The force required for debonding was calculated, and subsequently, the units were converted from Newton to megapascal (MPa).

## Results

**Figure 4:**
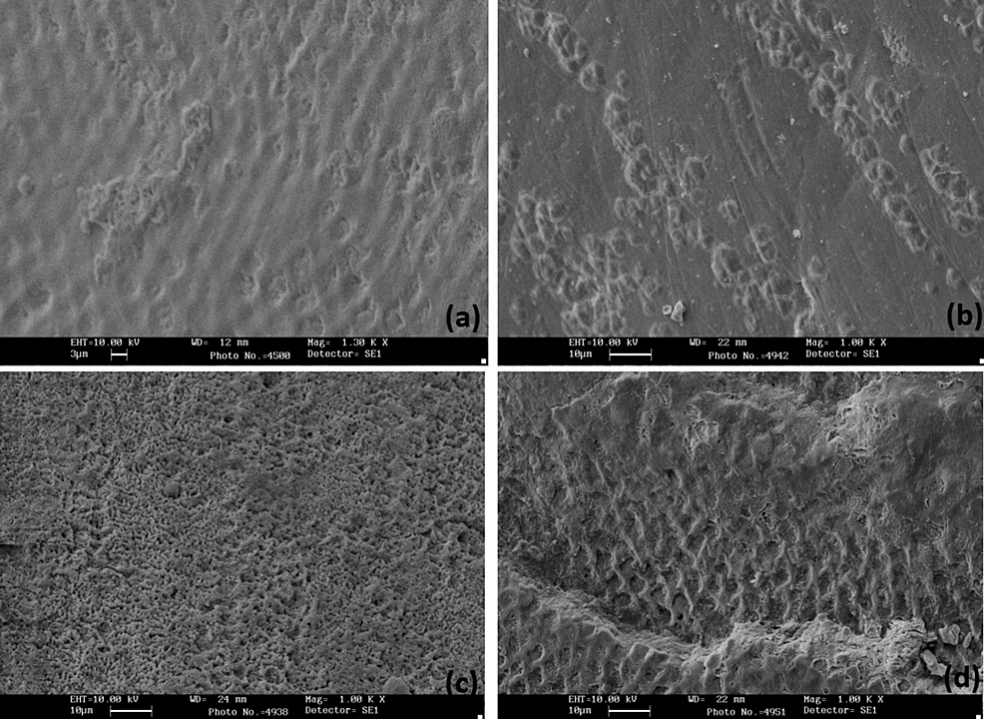
Scanning electron microscopic images showing the etching pattern on 1000× magnification (a) Control tooth and specimens treated with (b) citric acid, (c) phosphoric acid, and (d) hydrofluoric acid

SEM images after etching with citric acid, phosphoric acid, and hydrofluoric acid were evaluated at 1000× magnification with control. Type I etch was predominant in hydrofluoric acid, type II etch in phosphoric acid, and types IV and V in citric acid (Figure 4).

**Figure 5:**
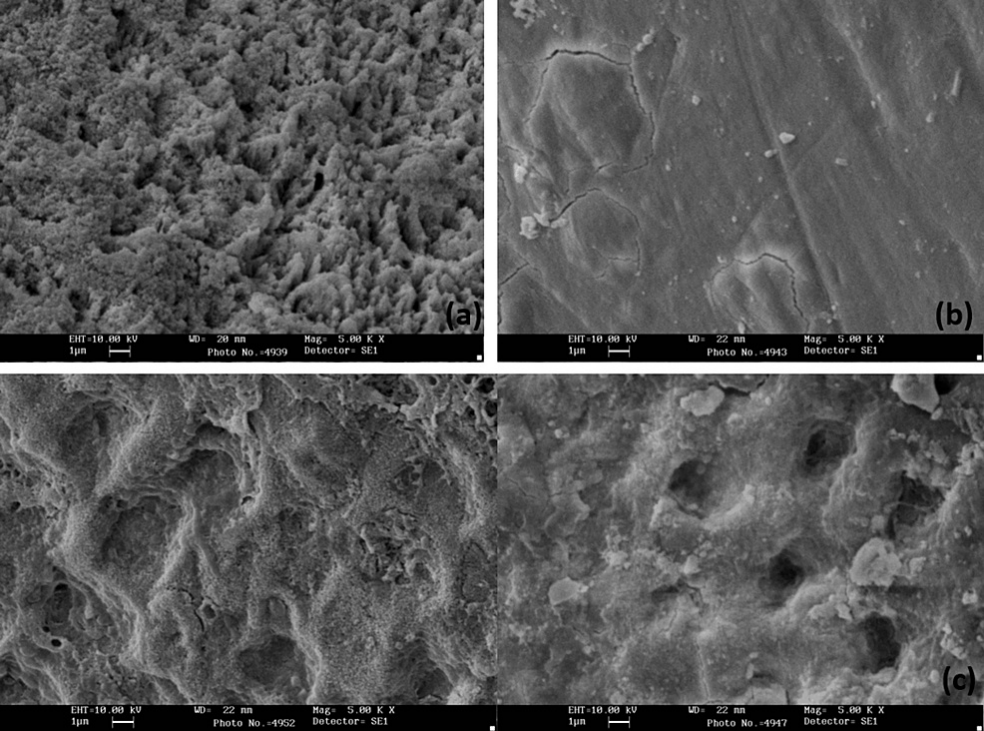
Scanning electron microscopic images showing etching patterns at 5000× magnification Specimens treated with (a) citric acid, (b) phosphoric acid, and (c) hydrofluoric acid

SEM images were evaluated after etching with phosphoric acid, citric acid, and hydrofluoric acid at 5000× magnification (Figure 5).

Fisher’s exact test showed a high statistical significance between the etching patterns of three etching agents (P < 0.001) (Table 1).

**Table 1:** Fisher’s exact test evaluation of difference in the etching patterns among the three etching agent groups df, degree of freedom *Statistical significance with P < 0.05

Etching patterns			
	Value	df	P-value
Fisher's exact test	60	8	<0.001*

**Figure 6:**
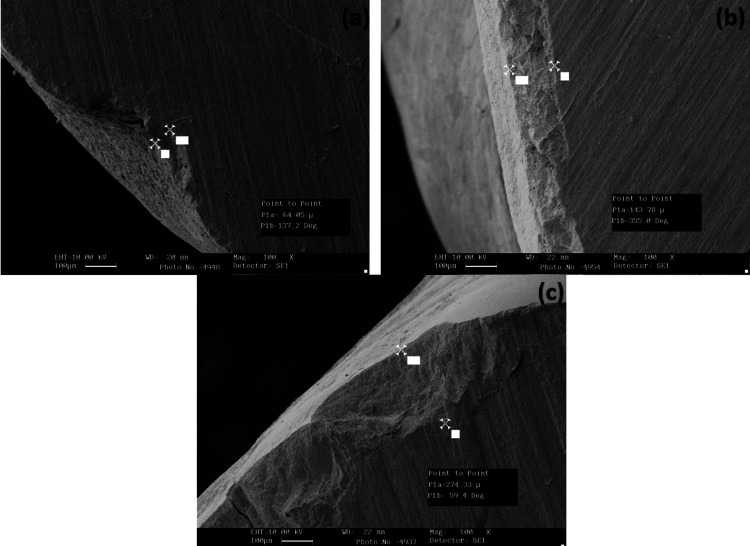
Scanning electron microscopic images showing the penetration depth of etchants among the samples at 100× magnification Specimens treated with (a) citric acid, (b) phosphoric acid, and (c) hydrofluoric acid

The teeth were etched with 20% citric acid, 37% phosphoric acid, and 11% hydrofluoric acid for 30 seconds. The surface profile measurements were carried out. With 11% hydrofluoric acid, the highest penetration depth of 276.47 µ was obtained, and with 20% citric Acid, the lowest penetration depth of 61.47 µ was obtained. Meanwhile, 37% phosphoric acid showed a 149.54 µ depth of penetration (Figure 6).

In the hydrofluoric acid group, the largest standard deviation (SD) was found. The smallest SD was in the group of citric acid. The maximum bond strength with a mean of 7.91 MPa was shown by hydrofluoric acid, followed by phosphoric acid at 4.84 MPa and citric acid at 2.43 MPa (Table 2).

**Table 2:** Variations between the etching depth of three groups using a one-way ANOVA test Groups: x = 20% citric acid, y = 37% phosphoric acid, and z = 11% hydrofluoric acid

Etching depth (µ)								
Groups	n	Mean (µ)	Std. deviation	Std. error	95% confidence interval for mean		Minimum	Maximum
					Lower bound	Upper bound		
x	10	61.47	5.16	1.630	57.78	65.16	50.74	67.02
y	10	149.54	10.68	3.377	141.91	157.18	130.86	170.64
z	10	276.47	12.24	3.870	267.71	285.22	260.54	299.64
Total	30	162.49	90.26	16.479	128.79	196.20	50.74	299.64

The post hoc Tukey HSD analysis showed no significant difference within groups. The specimens treated with hydrofluoric acid, phosphoric acid, and citric acid showed significant differences from the control group (P > 0.001) (Table 3).

**Table 3:** Analysis of variance for etching depth (µ) among the study groups Statistical significance of P < 0.05

Etching depth (µ)					
	Sum of squares	df	Mean square	F	ANOVA P-value
Between groups	233635.979	2	116817.989	1206.942	<0.001*
Within groups	2613.287	27	96.788		
Total	236249.265	29			

Intergroup correlations showed a high statistical correlation between etching depth & SBS, etching depth & etching pattern, and SBS & etching pattern between group A1, group A2, and group B (P ≤ 0.001) (Table 4).

**Table 4:** Statistical multiple comparisons of etching depth by the post hoc Tukey HSD test Statistical significance of P < 0.05

Groups		Mean difference	Std. error	P-value	95% confidence interval	
					Lower bound	Upper bound
20% citric acid	37% phosphoric acid	-88.08	4.40	<0.001*	-98.98	-77.17
20% citric acid	11% hydrofluoric acid	-215.00	4.40	<0.001*	-225.91	-204.09
37% phosphoric acid	11% hydrofluoric acid	-126.92	4.40	<0.001*	-137.83	-116.01

A positive statistical correlation was seen between etching depth & SBS. There was a negative correlation between etching depth & etching pattern and SBS & etching pattern.

Intra-group correlation showed a highly significant negative correlation between SBS & etching pattern (P = 0.42), whereas non-significant correlations were found between etching depth & SBS and etching depth & etching pattern within 20% citric acid etch group (P = 0.370 and 0.141, respectively) (Table 5).

**Table 5:** Variations between shear bond strength of four groups using the ANOVA test Groups: I = 20% citric acid, II = 37% phosphoric acid, III = 11% hydrofluoric acid, and IV = no etchant used (control) Statistical significance of P < 0.05

Shear bond strength					
	Sum of squares	df	Mean square	F	ANOVA P-value
Between groups	321.071	3	107.024	474.710	<0.001*
Within groups	8.116	36	0.225		
Total	329.187	39			

A non-significant correlation was found between each group of 37% phosphoric acid and 11% hydrofluoric acid within etching depth & SBS, etching depth & etching pattern, and SBS & etching pattern. Statistical comparison with the post hoc Tukey HSD test yielded significant results (Table 6).

**Table 6:** Statistical multiple comparisons of shear bond strength between groups using the post hoc Tukey HSD test Statistical significance of P < 0.05

Shear bond strength												
				Mean difference (I-J)		Std. error		P-value		95% confidence interval		
										Lower bound		Upper bound
20% citric acid (CA)		PA		-2.408		0.21234		<0.001*		-2.9799		-1.8361
		HF		-5.483		0.21234		<0.001*		-6.0549		-4.9111
		Control		2.1308		0.21234		<0.001*		1.5589		2.7027
37% phosphoric acid (PA)		HF		-3.075		0.21234		<0.001*		-3.6469		-2.5031
		Control		4.5388		0.21234		<0.001*		3.9669		5.1107
11% hydrofluoric acid (HF)		Control		7.6138		0.21234		<0.001*		7.0419		8.1857

Pearson’s correlation test among the different study groups yielded significant results (Table 7).

**Table 7:** Pearson's correlation test for groups A1, A2, and B Groups: A1 = etching pattern, A2 = etching depth (µ), and B = shear bond strength (MPa)

Pearson's correlation test				
Groups		N	Correlation	P-value
1	A2 & B	30	0.965	<0.001*
2	A1 & A2	30	-0.802	<0.001*
3	A1 & B	30	-0.790	<0.001*

## Discussion

Buonocore [2] advocated acid etching of enamel to improve adhesive substance retention, ushering in a new age. Silverstone et al. [5] researched and backed the method of acid etching. The etching procedure results in the disintegration of enamel material, creating an uneven enamel surface that makes it easier for an orthodontic attachment to be retained via its bonding agent. Etching and conditioning of tooth surfaces can enhance the micromechanical bond of adhesive restorative materials to the prepared tooth surfaces. The selective removal of component(s) from a solid surface can be done by etching. As a result, there is an increase in surface area, surface free energy, and wettability of the surface [11,12]. The chemical composition of tooth structure, the technique used, type, concentration, pH and viscosity of acid used, and time of application of acid are the various factors that influence the effects of acid etching. The present study has evaluated the effect of different chemical compositions of the etching agents for enamel conditioning [11,13]. We utilized 101 extracted premolars in this study. All the teeth samples were treated at the same time with three different etchants used in this study: 20% citric acid, 37% phosphoric acid, and 11% hydrofluoric acid. 

As the reported literature had conflicting results on acid solutions and their concentrations, we performed this study. The effectiveness of different etchants on the enamel surface was evaluated to study etching patterns and etching depth. Thirty-one (n = 30 and one control sample) samples were prepared for evaluation under the SEM to check the surface characteristics of etched enamel. Our findings were comparable to those of Parihar and Pilania [14]. It is reported in the literature [7] that the etching pattern was dependent mainly on the orientation of enamel crystals. “Honeycomb” topography resulted when the acid attack preceded in a direction parallel to the prism lines, causing preferential dissolution of core, with no effect on “borders” and “tails.” It can be hypothesized that if the etching is prolonged, then it affects the breakdown of the “honeycomb” structure as the penetration of the etchant may proceed perpendicular to the direction of prism lines. The difference in the loss of enamel structure was negligible when liquid and gel etchants were compared [15]. In fact, the development of acid gels was an improvement over the acid solution. The bond strength was greater when etched with acid solutions rather than with gel etchant [16,17].

Studies have reported that the application of 37% phosphoric acid for 30 seconds resulted in 16.4 µ penetration depth. Enamel tissue loss was found to be between 5 and 25 µm and at 9.9 µm when treated with 50% phosphoric acid. Phosphoric acid dissolved enamel at a faster rate when compared to citric acid [18]. As per Bhandari et al., the tag length observed under SEM varied from 5 to 12 µ [15]. When the acid was etched for 30 seconds, the observed tag length ranged from 7.47 ± 0.40 µ to 10 ± 0.64 µ, which aligns with reported literature [19]. In the present study, the least SBS was found in the control group, as there was no etching agent application. The mean SBS of 11% hydrofluoric acid had the highest bond strength (7.91 ± 0.36 MPa), followed by 37% phosphoric acid with a bond strength of 4.84 MPa. However, 20% citric acid showed the least bond strength (2.43 ± 0.28 MPa), and the difference between the groups was statistically significant.

Gupta et al. suggested that hydrofluoric acid could be a viable alternative to phosphoric acid for surface treatment in the repair of composite resin restorations [18]. Application of 37% phosphoric acid exhibited lower SBS when compared with 30% citric acid as a surface treatment. Our findings are in contrast to their study; however, the duration & concentration of application of citric acid seems to have caused this effect on enamel. The application of 50% citric acid for one minute did not lead to the attainment of sufficient bond strength, aligning with the findings of the present study. 

Multiple values have been proposed by various authors, and a maximum bond strength of 5.9-7.9 MPa (60-80 kg) is considered adequate [20]. The present study of the SBS of bonded brackets indicates a statistically significant difference when bonded using 11% hydrofluoric acid, 37% phosphoric acid, and 20% citric acid. Therefore, 11% hydrofluoric acid and 37% phosphoric acid showed clinically acceptable bond strength. In the current study, the application of hydrofluoric acid for 30 seconds on the substrate yielded the best outcome, while Lucena-Martín et al. utilized hydrofluoric acid for two minutes [19].

Raju et al. recommended a minimum bond strength of 7 MPa (75 kg) for sustainable bonding of orthodontic attachments [21]. The optimal force of translation with frequently used clinical orthodontic forces can range from 15 to 150 g. The forces of mastication are variable and may range up to 50 kg, which requires 4-5 MPa. Bonded brackets experience shear, tensile, and torsional forces in the oral cavity [21]. A statistically significant correlation between the etching pattern and SBS was found in the present study. Negative correlations were found between the etching depth and etching pattern and SBS with the etching pattern. It means that with an increase in the value of etching depth & SBS, the etching pattern value will be reduced [22-25]. Regular and distinct type I and type 2 patterns provided maximum adhesion, which coincides with the present study. 

In vitro SBS testing is a widely used method to assess the bond strength between orthodontic brackets and tooth enamel. In vitro testing cannot show variations in dynamic and static forces, as it is performed in a controlled laboratory environment. However, it has several limitations that restrict its ability to fully replicate the clinical scenario and predict bond strength in vivo.

## Conclusions

In vitro testing remains a valuable tool for initial screening and development of new orthodontic adhesives. We observed the highest penetration depth and maximum SBS with 11% hydrofluoric acid. However, it’s important to remember that the results should be interpreted with caution and not be taken as a direct prediction of clinical performance. Additional in vivo testing and long-term clinical studies are crucial for a more comprehensive evaluation of orthodontic adhesive bond strength.
